# Pseudophakic Cystoid Macular Oedema (PCME) Prevention in Patients with Non-Proliferative Diabetic Retinopathy (NPDR)—Randomized Controlled Trial

**DOI:** 10.3390/medicina58111667

**Published:** 2022-11-17

**Authors:** Anđela Jukić, Rajka Kasalica Žužul, Josip Pavan, Mila Lovrić, Ana Kozmar, Davor Plavec, Tomislav Kuzman, Miro Kalauz, Tomislav Jukić

**Affiliations:** 1 Department of Ophthalmology, University Hospital Dubrava, 10000 Zagreb, Croatia; 2 Clinical Institute of Laboratory Diagnosis, Clinical Hospital Centre, 10000 Zagreb, Croatia; 3 Children’s Hospital Srebrnjak, 10000 Zagreb, Croatia; 4 Clinic of Ophthalmology, Clinical Hospital Centre, 10000 Zagreb, Croatia

**Keywords:** pseudophakic cystoid macular oedema (PCME), central foveal subfield thickness (CFT), interleukin 6 (IL-6), non-proliferative diabetic retinopathy (NPDR)

## Abstract

*Background and Objectives:* The purpose of this study was to compare the effect of topical bromfenac and dexamethasone on the intraocular concentration of interleukin 6 (IL-6) and incidence of pseudophakic cystoid macular oedema (PCME) after cataract surgery in patients with non-proliferative diabetic retinopathy (NPDR). *Materials and Methods:* Ninety eyes of patients with mild-to-moderate NPDR that underwent phacoemulsification cataract surgery were divided into three groups. A detailed description of the clinical study protocol is described later in paper. In short, Group 1 received topical bromfenac (0.9 mg/mL), Group 2 dexamethasone (1 mg/mL), and Group 3 placebo, both preoperatively and postoperatively. Additionally, all patients received combined topical steroid and antibiotic drops (dexamethasone, neomycin and polymyxin B) 3 weeks postoperatively. On the day of the surgery, aqueous humour samples (0.1–0.2 mL) were obtained and IL-6 concentrations were analysed. Central foveal subfield thickness (CFT) measured using spectral-domain optical coherence tomography (SD-OCT) was analysed preoperatively and postoperatively. *Results:* There was no significant difference in IL-6 concentrations between groups. Postoperative CFT was significantly lower in the dexamethasone group compared to the placebo group. In addition, the correlation between IL-6 and CFT was statistically significant in the dexamethasone group. No patient developed PCME in any of the three groups. No adverse events were reported during the study. *Conclusion:* Topical bromfenac and dexamethasone have no significant effect on intraocular IL-6 concentration in patients with NPDR. Topical bromfenac is not more effective than topical dexamethasone in reducing postoperative CFT in patients with NPDR.

## 1. Introduction

The purpose of this study was to compare the effect of topical bromfenac and dexamethasone on the intraocular concentration of interleukin 6 (IL-6) and on the incidence of pseudophakic cystoid macular oedema (PCME) after cataract surgery in patients with non-proliferative diabetic retinopathy (NPDR). 

In patients with NPDR, no data comparing these commonly used drugs in ophthalmology are available and therefore it is reasonable to compare their effects in the targeted patient population.

Two clinical forms of postsurgical macular oedema can be present in patients with diabetes, alone or in combination: diabetic macular oedema (DME) and pseudophakic (Irvine–Gass) macular oedema (PCME) [1]. Both DME and PCME are characterised by the inflammatory breakdown of the blood–retinal barrier; however, different pathways are involved [2].

The diagnostic criteria for PCME are Inconsistent among published studies, resulting in large differences in the reported incidence [3]. The clinical definition of PCME on optical coherence tomography (OCT) differs among studies. Some researchers report a central foveal subfield thickness (CFT) increase of >40% and a decrease in macular sensitivity of >20% [4], while others report a CFT increase of >40 µm [5]. A few researchers indicate a CFT increase of >30% within 90 days after surgery [6,7], while others specify a CFT of >320 µm and visual acuity of 20/40 or worse [8]. Some researchers consider PCME as comprising a CFT increase of 10% from baseline [9], while others as a >35% increase in macular volume [10], or an increase of two standard deviations (SDs) above the preoperative mean CFT [11]. In addition, a >40% baseline CFT increase [3,12] and three SDs beyond the preoperative mean central macular thickness [13] were reported as PCME.

When analysed with OCT, PCME is characterised by retinal thickening, cystic hyporeflective lesions, and loss of foveal depression [12], showing a central, symmetric pattern. Transudate accumulates in small cysts in the inner nuclear layer, which may unite into larger cysts and reach the outer plexiform layer of the retina [14,15]. Munk et al. reported that PCME can be differentiated from DME based on SD-OCT [16]. Although some reports have shown that pre-existing DME is not essential for its postoperative occurrence, after cataract surgery PCME occurs mostly in patients with pre-existing DME [17]. 

Interleukin-6 (IL-6) is a cytokine associated with many intraocular inflammatory diseases, such as diabetic retinopathy (DR) [18]. IL-6 induces acute-phase reactions [19] and an ocular inflammatory response, frequently accompanied by the breakdown of the blood–ocular barriers [20], and it may play a central role in the development of inflammation after cataract surgery [21]. Several studies have investigated the connection between IL-6 and CFT in patients with diabetic retinopathy (DR). In a study by Funatsu et al., hypertension, aqueous levels of vascular endothelial growth factor (VEGF), and aqueous levels of IL-6 were associated with the exacerbation of macular oedema [22], and the levels of IL-6 correlated with the severity of DR and CFT, as measured using OCT [23,24,25]. Furthermore, in patients with DR, Klein et al. observed a positive correlation between IL-6 levels in the aqueous humour and total macular volume, as well as between IL-6 levels and CFT [26]. In addition, an association between elevated levels of IL-6 in the aqueous humour and the occurrence of ‘leakage’ in the macula on fluorescein angiography (FA) has been observed in patients with DM 6 months after cataract surgery [22]. Malecaze et al. found locally synthesised IL-6 as the main mediator of postoperative inflammation after cataract surgery and its concentrations in the aqueous humour increased significantly postoperatively [21]. Chu et al. found that the concentrations of IL-6 in the aqueous humour were significantly higher in patients who developed PCME [27]. 

In order to know the effect of a drug on IL-6 concentration in the aqueous humour, it may be better to take aqueous humour samples twice, before and after drug administration. Nevertheless, due to ethical reasons, cost and potential risk of infection, in our study aqueous humour samples were taken only once, after drug administration. The effects of topical bromfenac and dexamethasone on IL-6 concentration in the aqueous humour of patients with NPDR were determined by comparing concentrations between the experimental groups and between each experimental group and the placebo group. 

The effects of topical bromfenac and dexamethasone on IL-6 concentration in the aqueous humour of patients with NPDR are unknown and data presented here provide important new insights. 

In addition, the best anti-inflammatory prophylaxis for PCME in patients with NPDR is still unknown [28]. There are no published studies that have initiated corticosteroids preoperatively [29], however there are several studies that have favoured starting NSAID treatment preoperatively [12,13,15,30,31,32,33]. Diabetic retinopathy is considered a chronic subclinical inflammatory disease [34] and for patients with chronic non-infectious uveitis it is common to consider preoperative corticosteroids as prophylaxis for inflammation and CME [35]. Presently, there are no published studies with NPDR patients that explore the therapeutic effect of an NSAID in reducing PCME when compared directly with an equivalent dose of a corticosteroid. 

In this study, we hypothesised that topical bromfenac is more effective than topical dexamethasone in reducing IL-6 concentrations in the aqueous humour and in reducing the incidence of PCME in patients with NPDR. To directly compare the effect of topical NSAIDs and topical corticosteroids, both patient groups received therapy prior to surgery and postoperatively. 

All three study groups also received combined topical steroid and antibiotic drops postoperatively because most ophthalmologists believe that NSAIDs alone are ineffective in controlling excessive postoperative inflammation, which is expected in patients with risk factors like diabetes mellitus [29,36,37,38,39].

## 2. Materials and Methods

### Study Design

The study was randomized, double-blinded and vehicle-controlled. The study design has been adopted from a clinical trial that was conducted by our research group and it can be found on the ClinicalTrials.gov web site (https://clinicaltrials.gov/ct2/show/NCT04940338?cond=PCME&cntry=HR&city=Zagreb&draw=2&rank=1; 25 June 2021) using registration number NCT04940338. All procedures performed were in accordance with the ethical standards of the institutional research committee, with the 1964 Helsinki declaration and its later amendments or comparable ethical standards. The study protocol was approved by the Medical Research Ethics Committee of the Clinical Hospital Centre Zagreb (CR ZAG 02/21 AG 8.1-16/183-2). Informed consent was obtained from all individual participants included in the study.

The inclusion criteria involved patients with type II diabetes mellitus (DM), mild-to-moderate NPDR (EDTRS classification), and senile cataract grade II nuclear/cortical or posterior subcapsular (Lens Opacity Classification System III [LOCS III] [40]). All patients were scheduled for cataract surgery with posterior chamber intraocular lens (IOL) implantation. Nuclear opalescence was graded according to the LOCS III [40]. The exclusion criteria were anterior segment pathology (pseudoexfoliation syndrome, corneal opacities); posterior segment pathology (DME, previous DME treatment, previous retinal photocoagulation therapy, age-related macular degeneration, retinal vascular diseases or history of uveitis); intraoperative complications (posterior capsular rupture, vitreous loss, IOL not implanted in the capsular bag); postoperative complications (leaking incision, increased intraocular pressure, corneal oedema or inflammation); therapy for glaucoma; patients on antihypertensive therapy; topical or systemic NSAIDs or steroids; previous steroid responders or hypersensitivity to the NSAID drug class; and previous ocular trauma or intraocular surgery. 

**Figure 1:**
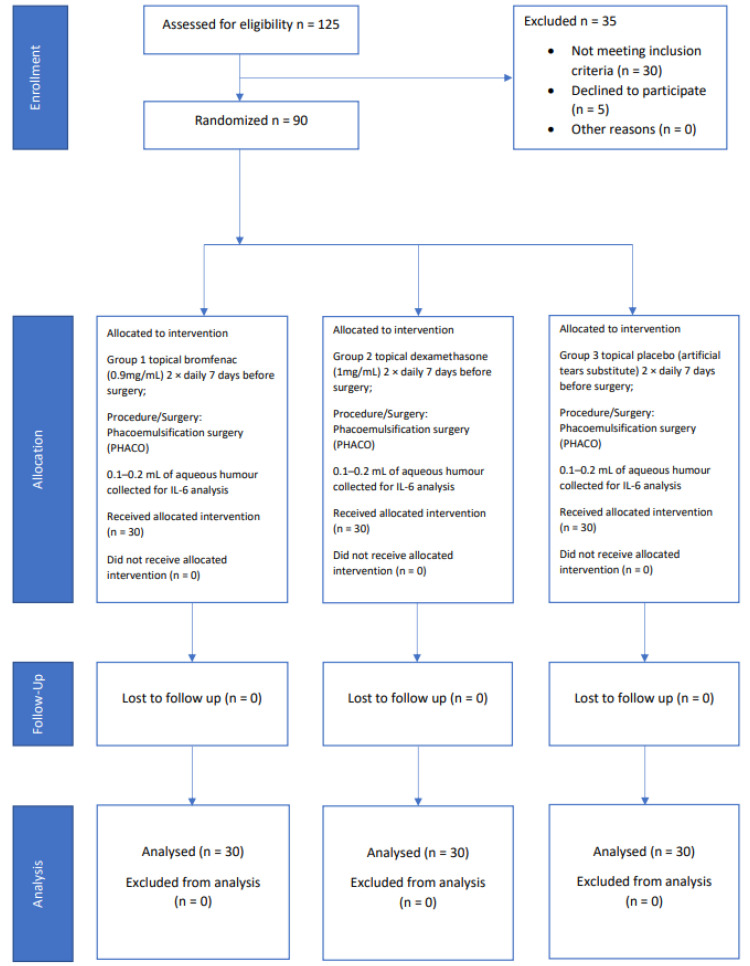
CONSORT flow diagram.

A total of 125 patients with NPDR referred for cataract surgery were assessed for eligibility, as shown in the CONSORT flow diagram (Figure 1). After exclusion, ninety eyes from 90 patients were randomized in the study (30 per each group). The patients were randomized into three groups and the study drugs were masked for both the patients and examiners. Random allocation was made in blocks of nine patients, three in each group. Drug pipettes were covered with tape by the hospital pharmacy and placed in marked envelopes. Blinding was revealed after the data were analysed. Group 1 received topical bromfenac (0.9 mg/mL) twice daily, Group 2 received topical dexamethasone (1 mg/mL) twice daily and Group 3 received topical placebo (artificial tear substitute) twice daily, for 7 days preoperatively and 3 weeks postoperatively. Macular oedema was defined as a CFT increase of 40% from the baseline [3,12]. Approximately 0.1–0.2 mL of the aqueous humour was collected at the beginning of the surgery through a paracentesis, transported to the laboratory in dry ice with a dedicated box, and stored at −80 °C until analysis. IL-6 concentration was analysed using the Human IL-6 Quantikine ELISA kit R&D Systems, Bio-Techne Corporation, Minneapolis, Minnesota, USA.

CFT was measured using a spectral-domain OCT device 7 days prior to surgery, on the day of the surgery and on postoperative day 1, 7, 30 and 90. The macular thickness was reported based on the ETDRS thickness map. CFT was defined as the mean macular thickness in the central 1.0 mm area. All patients were examined by a single researcher to limit observer bias. One experienced surgeon performed all surgical procedures, which consisted of topical anaesthesia, clear corneal small incision, capsulorhexis and phacoemulsification with the implantation of a foldable hydrophobic acrylic intraocular lens. At the end of the surgery, cefuroxime (1 mg) was instilled into the anterior chamber. Postoperatively, topical steroid antibiotic drops (dexamethasone, neomycin and polymyxin B, 1 mg, 3500 IU and 6000 IU/mL, respectively) were prescribed four times daily for a week and then tapered over the next 3 weeks (three times daily for a week, then two times daily for a week and then one time daily for a week). Information on adverse events was collected for all patients after the first administration of the study drug on day −7 and continuing until day 90.

## 3. Statistical Analysis 

Statistical power analysis indicated that, with CFT as the primary outcome, the overall sample size required in this study was 90 patients. A priori statistical power analysis was performed using the GPower software (G*Power 3.1.9.7), Faul, Erdfelder, Lang, & Buchner, 2007 [41,42] where the statistical power (1−β) was set at 0.80, α was set at 0.05 (two-tailed test), and the effect size was set at *d* = 0.30.

One-way analysis of variance (ANOVA) was used to examine the differences between the three groups of patients with respect to intraocular IL-6 concentrations. The condition of homogeneity of variance was achieved. Statistical significance was set at *p* < 0.05. 

Analysis of covariance and control of initial CFT measurements before the surgical procedure was used to examine the effect of topical NSAIDs and topical corticosteroids on the incidence of pseudophakic cystoid macular oedema. An experimental 3 × 5 design with repeated measurements of the second variable was used. The first variable (administration of a different drug) had three levels (three groups, each with 30 patients), whereas the second variable had five levels (five measurement points; every measurement was carried out on all patients). Control of the measurement starting point (7 days before the surgical procedure) was performed because of the initial differences in CFT measurements. The correlation between IL-6 levels, CFT, and CFT changes was analysed using Spearman’s rank correlation.

## 4. Results

### 4.1. The Effect of Topical Bromfenac and Dexamethasone on the Incidence of Pseudophakic Cystoid Macular Oedema

**Figure 2:**
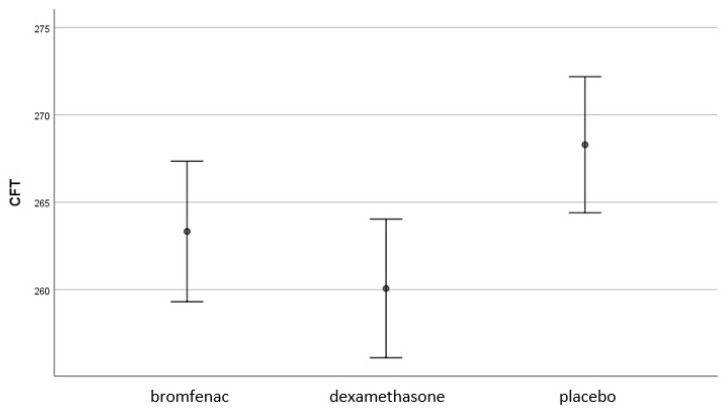
CFT (µm) was significantly lower in experimental Group 2 (topical dexamethasone) than in the placebo group 90 days after the surgery (*F* (2.86) = 4.46, *p* < 0.014). Differences in CFT between experimental Group 1 (topical bromfenac) and the placebo group, as well as between the two experimental groups, were not statistically significant; Bromfenac group *n*= 30; Dexamethasone group *n*= 30; Placebo group *n* = 30; CFT, central foveal subfield thickness.

No patient developed PCME in any of the three groups for the 90 days of the study. Contrast analysis indicated that 90 days after the surgery, CFT was significantly lower in experimental Group 2 (topical dexamethasone) than in the placebo group (*F* (2.86) = 4.46, *p* < 0.014). Differences in CFT between experimental Group 1 (topical bromfenac) and placebo group, as well as between the two experimental groups, were not statistically significant (Figure 2). 

**Figure 3:**
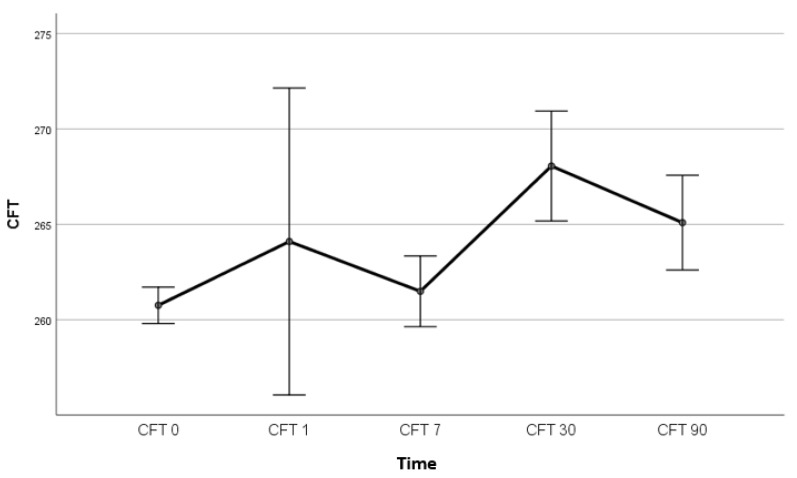
CFT (µm) peaked one month after surgery, but the difference was not statistically significant (*F* (1.264, 108.75) = 0.498, *p* = 0.525); *n* = 90; CFT, central foveal subfield thickness; CFT 0, central foveal subfield thickness on the day of the surgery; CFT 1, central foveal subfield thickness on first postoperative day; CFT 7, central foveal subfield thickness seven days after surgery; CFT 30, central foveal subfield thickness 30 days after surgery; CFT 90, central foveal subfield thickness 90 days after surgery.

Macular thickness peaked one month after surgery, which is consistent with previous studies [4,43]; however, the difference was not statistically significant (*F* (1.264, 108.75) = 0.498, *p* = 0.525) (Figure 3).

**Figure 4:**
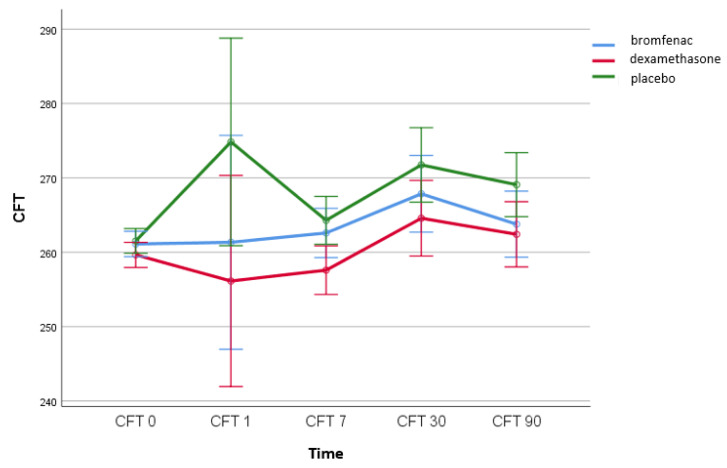
No interaction effect was observed between the time of CFT measurement (µm) and different groups (*F* (2.53, 108.74) = 1.032, *p* = 0.373); bromfenac group *n* = 30; dexamethasone group *n* = 30; placebo group *n*= 30; CFT, central foveal subfield thickness; CFT 0, central foveal subfield thickness on the day of the surgery; CFT 1, central foveal subfield thickness on first postoperative day; CFT 7, central foveal subfield thickness seven days after surgery; CFT 30, central foveal subfield thickness 30 days after surgery; CFT 90, central foveal subfield thickness 90 days after surgery.

Differences in central foveal subfield thickness with respect to the time of measurement and the type of drug/group are shown in Figure 4. No interaction effect was observed between the time of central retinal thickness measurement and different groups (bromfenac group, dexamethasone group, placebo group) (*F* (2.53, 108.74) = 1.032, *p* = 0.373) (Figure 4).

### 4.2. The Correlation between IL-6 Levels and CFT

The correlation between IL-6 levels and CFT was analysed using Spearman’s rank correlation. In the group treated with dexamethasone, there was a statistically significant correlation between IL-6 levels and CFT values measured on the 1st, 7th and 30th day following the surgical procedure, and between IL-6 values and change in CFT on the 30th and 90th day following the surgical procedure when compared with the measurement conducted 7 days before the surgical procedure (Table 1 and Table 2). Correlation levels ranged from weak to strongly positive. In the group treated with bromfenac and in the placebo group, there was no statistically significant correlation between IL-6 levels, CFT, and change in CFT when compared to the measurement conducted 7 days before the surgical procedure.

### 4.3. The Effect of Topical Bromfenac and Dexamethasone on Intraocular Interleukin-6 (IL-6) Concentrations

**Figure 5:**
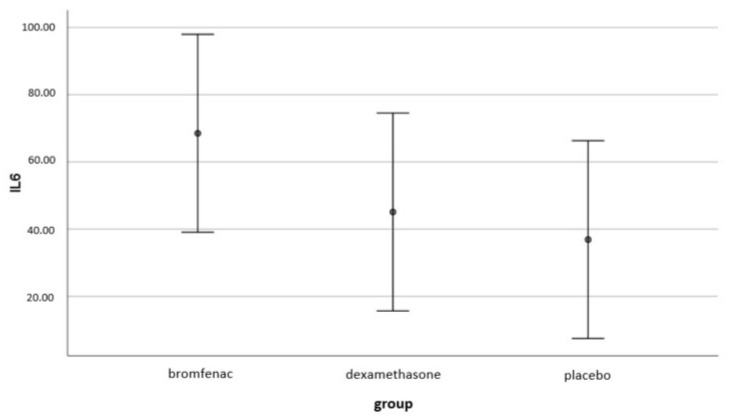
Difference in IL-6 concentration (pg/mL) between the groups was not statistically significant (*F* (2, 87) = 1.227, *p* < 0.298); bromfenac group *n* = 30; dexamethasone group *n*= 30; placebo group *n* = 30.

The results indicate that there is no statistically significant difference between the three groups with respect to intraocular IL-6 concentration (*F* (2, 87) = 1.227, *p* < 0.298). (Figure 5). 

### 4.4. Differences in Age and in DM Duration (Risk Factors for PCME)

There were no statistically significant differences in age or DM duration between the groups.

### 4.5. Intraocular Pressure (IOP) and Best-Corrected Visual Acuity (BCVA)

IOP was similar in all patients preoperatively and postoperatively, and no patient required additional postoperative treatment for elevated IOP. In all treatment groups, best-corrected visual acuity improved rapidly over the first week after surgery, and the improvements were sustained over 90 days.

No adverse events were reported during the study.

## 5. Discussion

The results of this study indicate that changes in CFT occurred under the influence of different types of treatment drugs, and CFT was significantly lower in the dexamethasone group compared to the placebo group. No statistically significant differences in CFT were observed between other compared patient groups. There were no statistically significant differences in age or DM duration between the groups, indicating that patient age and DM duration do not play a role in the response to the different therapeutic agents used in our study.

The analysis reveals no statistically significant differences in the IL-6 concentrations in the aqueous humour between different groups, which could mean that topical bromfenac and topical dexamethasone have no significant effect on intraocular IL-6 concentration in patients with NPDR. This result should be further investigated. In the group treated with dexamethasone, there was a statistically significant (mild to strong positive) correlation between IL-6 levels and postoperative CFT values when compared with the measurement conducted 7 days before the surgical procedure. Interestingly, in the placebo group, correlations that were not statistically significant were inversely proportional, unlike those in the group treated with bromfenac (in which the correlation was almost non-existent), which may indicate different types of drug interactions on molecular mechanisms affecting the relationship between IL-6 and CFT. 

Our results are supportive of data reported by Matsumura et al., who investigated the effect of topical bromfenac 0.1% and topical 0.02% fluorometholone on the concentration of IL-6 in the aqueous humour of patients after cataract surgery and found that there was no significant difference in IL-6 concentration between groups [30].

For PCME prevention, some researchers believe that NSAIDs are necessary in high-risk cases, such as in patients with DR [28,31]. In a retrospective study conducted by Modjtahedi et al. with NPDR patients, there was no difference in PCME incidence after cataract surgery regardless of whether NSAID therapy was used perioperatively [4]. To date, several studies on PCME prevention that indicate a beneficial therapeutic effect of the combination of NSAIDs and corticosteroids in patients with NPDR compared to corticosteroid monotherapy have been published, as shown in Table 3.

In these studies, groups of patients who received topical NSAIDs received therapy both preoperatively and postoperatively; in contrast, those who received topical corticosteroids received this therapy only postoperatively, leading to large differences in dosage. Therefore, in order to compare the results, in this study both groups received therapy both preoperatively and postoperatively. 

Corticosteroids have far wider anti-inflammatory properties, and it is difficult to explain the greater therapeutic effect of NSAIDs in reducing PCME compared with an equivalent dose of corticosteroids [45]. The advantages of NSAIDs over corticosteroids include a reduced risk of secondary infections, good IOP control and additional analgesic effects [46]. An additional reason for NSAID use is their ability to reduce surgical miosis and pain after cataract surgery. However, some studies indicate that corticosteroids provide similar therapeutic benefits [47,48].

The American Academy of Ophthalmology (AAO) Ophthalmic Technology Assessment Committee Retina/Vitreous Panel reported that NSAID therapy is successful in reducing PCME and may accelerate visual recovery after surgery. Nevertheless, NSAIDs do not modify long-term visual outcomes (>3 months), and the benefits of NSAID therapy can be achieved with equivalent doses of corticosteroids. In addition, the synergistic effect of NSAIDs and corticosteroids seems questionable regarding their overlapping mechanisms of action [49], and topical NSAIDs used simultaneously with topical corticosteroids may increase the potential for slow or delayed healing. Therefore, their combined use may result in keratitis in susceptible patients, resulting in epithelial breakdown, corneal thinning, erosion, ulceration, or perforation [36]. The reported prevalence of diabetic keratopathy (superficial punctate epitheliopathy, persistent epithelial erosions, corneal oedema, and corneal hypoesthesia) is around 70% among diabetic patients [50]. 

Most ophthalmologists widely accept that NSAIDs alone are ineffective in controlling excessive postoperative inflammation and are rarely used without corticosteroids postoperatively, especially in patients with diabetes mellitus, PEX syndrome or uveitis [29,36,37,38,39]. The question remains whether the risk for PCME compensates for the risk of adverse effects resulting from their concomitant use. 

Our results indicated that application of both NSAIDs and corticosteroids for PCME prevention in patients with mild-to-moderate NPDR seems to be unnecessary. Further research should explore the optimal route and dosage of corticosteroid treatment and its long-term benefits [51].

The strength of our study is that this is the first prospective study to examine the effect of preoperative topical bromfenac and topical dexamethasone on IL-6 concentration in the aqueous humour of patients with mild-to-moderate NPDR and correlate these concentrations with the development of postoperative macular changes. In addition, this is the first prospective study to explore the therapeutic effect of NSAIDs in reducing PCME when compared directly with an equivalent dose of a corticosteroid.

A limitation of this study is the absence of data on the duration of surgery or phacoemulsification-related variables. Other limitations were the small sample size and lack of long-term follow-up (>3 months). Furthermore, data were not collected on HbA1c levels, which is otherwise an important risk factor for the development of PCME. In addition, a limitation of the study was the fact that aqueous humour samples were collected only once, after drug administration. Our future research will address some of the still unsolved issues on this topic and it might provide additional valuable data.

## 6. Conclusions

Our results indicate that topical bromfenac and dexamethasone did not significantly affect intraocular IL-6 concentration in patients with NPDR. Topical bromfenac did not show more efficacy than topical dexamethasone in reducing postoperative CFT in patients with NPDR. Patients treated with topical dexamethasone showed statistically significant differences regarding postoperative CFT compared to the placebo group. This result should be further investigated to clarify the role of preoperative dexamethasone application and higher postoperative doses, and to establish the role of topical bromfenac in preventing PCME in patients with NPDR.

## Figures and Tables

**Table 1:** Correlation between IL-6 levels (pg/mL) and CFT values (µm) in the group treated with dexamethasone.

Pair of Variables	Dexamethasone			
	Valid N	Spearman R	t(N-2)	*p*-Value
IL6 & CFT -7	30	0.315	1.755	0.090
IL6 & CFT 0	30	0.265	1.455	0.157
IL6 & CFT 1	30	0.370	2.110	0.044
IL6 & CFT 7	30	0.376	2.148	0.040
IL6 & CFT 30	30	0.465	2.778	0.010
IL6 & CFT 90	30	0.340	1.911	0.066

**Table 2:** Correlation between IL-6 levels (pg/mL) and change in CFT values (µm) in the group treated with dexamethasone.

Pair of Variables	Dexamethasone			
	Valid N	Spearman R	t(N-2)	*p*-Value
IL6 & CFT 0	30	0.171	0.920	0.366
IL6 & CFT 1	30	0.309	1.718	0.097
IL6 & CFT 7	30	0.170	0.913	0.369
IL6 & CFT 30	30	0.609	4.064	0.000
IL6 & CFT 90	30	0.465	2.781	0.010
IL6 & CFT 0	30	0.171	0.920	0.366

**Table 3:** PCME prevention studies comparing the combination of NSAIDs and corticosteroids with corticosteroid monotherapy in NPDR patients.

Pollack 2017 [7]	Nepafenac 0.1%+ Dexamethasone 0.1%	Dexamethasone 0.1%	*p* = 0.01
Singh 2012 [6]	Nepafenac 0.1%+ prednisolone	Prednisolone (Omnipred)	*p* < 0.001
Singh 2017 [32]	Nepafenac 0.3%+ prednisolone	Prednisolone (Omnipred)	*p* < 0.001
Singh 2017 [33]	Nepafenac 0.1%+ prednisolone	Prednisolone (Omnipred)	*p* = 0.001; *p* = 0.018
Sarfraz 2017 [9]	Nepafenac 0.1%+ prednisolone 0.1%	1% Prednisolone Acetate	*p* < 0.05
Entezari 2017 [44]	Diclofenac 0.1%+ corticosteroid	Corticosteroid (not specified)	*p* = 0.003

## Data Availability

The data that support the findings of this study are available from the corresponding author, A.J., upon reasonable request.

## References

[1] Baker C.W., Almukhtar T., Bressler N.M., Glassman A.R., Grover S., Kim S.J., Murtha T.J., Rauser M.E., Stockdale C., Diabetic Retinopathy Clinical Research Network Authors/Writing Committee (2013). Macular edema after cataract surgery in eyes without preoperative central-involved diabetic macular edema. JAMA Ophthalmol..

[2] Modjtahedi B.S., Paschal J.F., Batech M., Luong T.Q., Fong D.S. (2017). Perioperative Topical Nonsteroidal Anti-inflammatory Drugs for Macular Edema Prophylaxis Following Cataract Surgery. Am. J. Ophthalmol..

[3] Kim S.J., Belair M.-L., Bressler N.M., Dunn J.P., Thorne J.E., Kedhar S.R., Jabs D.A. (2008). A method of reporting macular edema after cataract surgery using optical coherence tomography. Retina.

[4] Yang J., Cai L., Sun Z., Ye H., Fan Q., Zhang K., Lu W., Lu Y. (2017). Risk factors for and diagnosis of pseudophakic cystoid macular edema after cataract surgery in diabetic patients. J. Cataract Refract. Surg..

[5] Ramakrishnan S., Baskaran P., Talwar B., Venkatesh R. (2015). Prospective, Randomized Study Comparing the Effect of 0.1% Nepafenac and 0.4% Ketorolac Tromethamine on Macular Thickness in Cataract Surgery Patients With Low Risk for Cystoid Macular Edema. Asia Pac. J. Ophthalmol..

[6] Singh R., Alpern L., Jaffe G.J., Lehmann R.P., Lim J., Reiser H.J., Sall K., Walters T., Sager D. (2012). Evaluation of nepafenac in prevention of macular edema following cataract surgery in patients with diabetic retinopathy. Clin. Ophthalmol..

[7] Pollack A., Staurenghi G., Sager D., Mukesh B., Reiser H., Singh R.P. (2017). Prospective randomised clinical trial to evaluate the safety and efficacy of nepafenac 0.1% treatment for the prevention of macular oedema associated with cataract surgery in patients with diabetic retinopathy. Br. J. Ophthalmol..

[8] Shorstein N.H., Liu L., Waxman M.D., Herrinton L.J. (2015). Comparative Effectiveness of Three Prophylactic Strategies to Prevent Clinical Macular Edema after Phacoemulsification Surgery. Ophthalmology.

[9] Sarfraz M.H., Haq R.I.U., Mehboob M.A. (2017). Effect of topical nepafenac in prevention of macular edema after cataract surgery in patients with non-proliferative diabetic retinopathy. Pak. J. Med. Sci..

[10] McCafferty S., Harris A., Kew C., Kassm T., Lane L., Levine J., Raven M. (2017). Pseudophakic cystoid macular edema prevention and risk factors; prospective study with adjunctive once daily topical nepafenac 0.3% versus placebo. BMC Ophthalmol..

[11] Perente I., Utine C.A., Ozturker C., Cakir M., Kaya V., Eren H., Kapran Z., Yilmaz O.F. (2007). Evaluation of macular changes after uncomplicated phacoemulsification surgery by optical coherence tomography. Curr. Eye Res..

[12] Yonekawa Y., Kim I.K. (2012). Pseudophakic cystoid macular edema. Curr. Opin. Ophthalmol..

[13] Kusbeci T., Eryigit L., Yavaş G., Inan U.U. (2012). Evaluation of cystoid macular edema using optical coherence tomography and fundus fluorescein angiography after uncomplicated phacoemulsification surgery. Curr. Eye Res..

[14] Sigler E.J., Randolph J.C., Kiernan D.F. (2016). Longitudinal analysis of the structural pattern of pseudophakic cystoid macular edema using multimodal imaging. Graefes. Arch. Clin. Exp. Ophthalmol..

[15] Flach A.J. (1998). The incidence, pathogenesis and treatment of cystoid macular edema following cataract surgery. Trans. Am. Ophthalmol. Soc..

[16] Munk M.R., Jampol L.M., Simader C., Huf W., Mittermüller T.J., Jaffe G.J., Schmidt-Erfurth U. (2015). Differentiation of Diabetic Macular Edema From Pseudophakic Cystoid Macular Edema by Spectral-Domain Optical Coherence Tomography. Investig. Ophthalmol. Vis. Sci..

[17] Kim S.J., Equi R., Bressler N.M. (2007). Analysis of macular edema after cataract surgery in patients with diabetes using optical coherence tomography. Ophthalmology.

[18] Yao Y., Li R., Du J., Long L., Li X., Luo N. (2019). Interleukin-6 and Diabetic Retinopathy: A Systematic Review and Meta-Analysis. Curr. Eye Res..

[19] Funatsu H., Yamashita H., Ikeda T., Mimura T., Eguchi S., Hori S. (2003). Vitreous levels of interleukin-6 and vascular endothelial growth factor are related to diabetic macular edema. Ophthalmology.

[20] Hoekzema R., Verhagen C., van Haren M., Kijlstra A. (1992). Endotoxin-induced uveitis in the rat. The significance of intraocular interleukin-6. Investig. Ophthalmol. Vis. Sci..

[21] Malecaze F., Chollet P., Cavrois E., Vita N., Arné J.L., Ferrara P. (1991). Role of interleukin 6 in the inflammatory response after cataract surgery. An experimental and clinical study. Arch. Ophthalmol..

[22] Funatsu H., Yamashita H., Noma H., Shimizu E., Mimura T., Hori S. (2002). Prediction of macular edema exacerbation after phacoemulsification in patients with nonproliferative diabetic retinopathy. J. Cataract. Refract. Surg..

[23] Funatsu H., Yamashita H., Noma H., Mimura T., Yamashita T., Hori S. (2002). Increased levels of vascular endothelial growth factor and interleukin-6 in the aqueous humor of diabetics with macular edema. Am. J. Ophthalmol..

[24] Funatsu H., Noma H., Mimura T., Eguchi S., Hori S. (2009). Association of vitreous inflammatory factors with diabetic macular edema. Ophthalmology.

[25] Oh I.K., Kim S.-W., Oh J., Lee T.S., Huh K. (2010). Inflammatory and angiogenic factors in the aqueous humor and the relationship to diabetic retinopathy. Curr. Eye Res..

[26] Klein R., Klein B.E.K., Knudtson M.D., Wong T.Y., Tsai M.Y. (2006). Are inflammatory factors related to retinal vessel caliber? The Beaver Dam Eye Study. Arch. Ophthalmol..

[27] Chu L., Wang B., Xu B., Dong N. (2013). Aqueous cytokines as predictors of macular edema in non-diabetic patients following uncomplicated phacoemulsification cataract surgery. Mol. Vis..

[28] Laursen S.B., Erichsen J.H., Holm L.M., Kessel L. (2019). Prevention of macular edema in patients with diabetes after cataract surgery. J. Cataract Refract. Surg..

[29] Alnagdy A.A., Abouelkheir H.Y., El-Khouly S.E., Tarshouby S.M. (2018). Impact of topical nonsteroidal anti-inflammatory drugs in prevention of macular edema following cataract surgery in diabetic patients. Int. J. Ophthalmol..

[30] Matsumura T., Iwasaki K., Arimura S., Takeda R., Takamura Y., Inatani M. (2021). Topical bromfenac reduces multiple inflammatory cytokines in the aqueous humour of pseudophakic patients. Sci. Rep..

[31] Olson R.J., Braga-Mele R., Chen S.H., Miller K.M., Pineda R., Tweeten J.P., Musch D.C. (2017). Cataract in the Adult Eye Preferred Practice Pattern®. Ophthalmology.

[32] Singh R.P., Lehmann R., Martel J., Jong K., Pollack A., Tsorbatzoglou A., Staurenghi G., Cervantes G.C.-C., Alpern L., Modi S. (2017). Nepafenac 0.3% after Cataract Surgery in Patients with Diabetic Retinopathy: Results of 2 Randomized Phase 3 Studies. Ophthalmology.

[33] Singh R.P., Staurenghi G., Pollack A., Adewale A., Walker T.M., Sager D., Lehmann R. (2017). Efficacy of nepafenac ophthalmic suspension 0.1% in improving clinical outcomes following cataract surgery in patients with diabetes: An analysis of two randomized studies. Clin. Ophthalmol..

[34] Dong N., Xu B., Wang B., Chu L. (2013). Study of 27 aqueous humor cytokines in patients with type 2 diabetes with or without retinopathy. Mol. Vis..

[35] Chen J.L., Bhat P., Lobo-Chan A.-M. (2019). Perioperative Management of Uveitic Cataracts. Adv. Ophthalmol. Optom..

[36] Gaynes B.I., Onyekwuluje A. (2008). Topical ophthalmic NSAIDs: A discussion with focus on nepafenac ophthalmic suspension. Clin. Ophthalmol..

[37] Kim S.J., Flach A.J., Jampol L.M. (2010). Nonsteroidal anti-inflammatory drugs in ophthalmology. Surv. Ophthalmol..

[38] (2012). Leamingsurveys. Survey of US ASCRS Members. https://bmedesign.engr.wisc.edu/projects/f11/balance_trainer/file/view/c3bea4e1-e005-4c5e-ae27-ebc892a30e17/WBB_Final_Paper.pdf.

[39] Karkhur S., Verma V., Gupta R., Sharma B. (2022). Commentary: Newer non-steroidal anti-inflammatory drugs for postoperative management in phacoemulsification. Are topical corticosteroids still obligatory?. Indian J. Ophthalmol..

[40] Chylack L.T., Wolfe J.K., Singer D.M., Leske M.C., Bullimore M.A., Bailey I.L., Friend J., McCarthy D., Wu S.Y. (1993). The Lens Opacities Classification System III. The Longitudinal Study of Cataract Study Group. Arch. Ophthalmol..

[41] Faul F., Erdfelder E., Lang A.G., Buchner A. (2007). G*Power 3: A flexible statistical power analysis program for the social, behavioral, and biomedical sciences. Behav. Res. Methods.

[42] Faul F., Erdfelder E., Buchner A., Lang A.G. (2009). Statistical power analyses using G*Power 3.1: Tests for correlation and regression analyses. Behav. Res. Methods.

[43] Zur D., Loewenstein A. (2017). Postsurgical Cystoid Macular Edema. Dev. Ophthalmol..

[44] Entezari M., Ramezani A., Nikkhah H., Yaseri M. (2017). The effect of topical sodium diclofenac on macular thickness in diabetic eyes after phacoemulsification: A randomized controlled trial. Int. Ophthalmol..

[45] Kim S.J., Patel S.N., Sternberg P. (2016). Routine Use of Nonsteroidal Anti-inflammatory Drugs with Corticosteroids in Cataract Surgery: Beneficial or Redundant?. Ophthalmology.

[46] Rossetti L., Bujtar E., Castoldi D., Torrazza C., Orzalesi N. (1996). Effectiveness of diclofenac eyedrops in reducing inflammation and the incidence of cystoid macular edema after cataract surgery. J. Cataract Refract. Surg..

[47] Zanetti F.R., Fulco E.A.M., Chaves F.R.P., da Costa Pinto A.P., Arieta C.E.L., Lira R.P.C. (2012). Effect of preoperative use of topical prednisolone acetate, ketorolac tromethamine, nepafenac and placebo, on the maintenance of intraoperative mydriasis during cataract surgery: A randomized trial. Indian J. Ophthalmol..

[48] Simone J.N., Pendelton R.A., Jenkins J.E. (1999). Comparison of the efficacy and safety of ketorolac tromethamine 0.5% and prednisolone acetate 1% after cataract surgery. J. Cataract. Refract. Surg..

[49] Schoenberger S.D., Kim S.J. (2013). Nonsteroidal anti-inflammatory drugs for retinal disease. Int. J. Inflamm..

[50] Schultz R.O., Van Horn D.L., Peters M.A., Klewin K.M., Schutten W.H. (1981). Diabetic keratopathy. Trans. Am. Ophthalmol. Soc..

[51] Wielders L.H.P., Schouten J.S.A.G., Winkens B., van den Biggelaar F.J.H.M., Veldhuizen C.A., Murta J.C.N., Goslings W.R.O., Kohnen T., Tassignon M.-J., Joosse M.V. (2018). Randomized controlled European multicenter trial on the prevention of cystoid macular edema after cataract surgery in diabetics: ESCRS PREMED Study Report 2. J. Cataract Refract. Surg..

